# Free Radical Scavenging Activity of Leaves of *Alocasia indica* (Linn)

**DOI:** 10.4103/0250-474X.56036

**Published:** 2009

**Authors:** W. A. Mulla, V. R. Salunkhe, S. B. Kuchekar, M. N. Qureshi

**Affiliations:** Government College of Pharmacy, Vidyanagar, Karad-415 124, India; 1 AISSMS College of Pharmacy, Pune-415 005, India; 2 AIT Institute of Pharmacy, Malegaon-423 203, Nasik, India

**Keywords:** Antioxidant, free radicals, *Alocasia indicia*, lipid peroxidation, ascorbic acid

## Abstract

The free radical scavenging potential of the plant *Alocasia indica*(Linn.) was studied by using different antioxidant models of screening like scavenging of 1,1-diphenyl-2-picryl hydrazyl radical, nitric oxide radical, superoxide anion radical, hydroxyl radical, iron chelating activity, total antioxidant capacity, non-enzymatic glycosylation of haemoglobin, rapid screening for antioxidant compounds by thin layer chromatography. The hydroalcoholic extract at 1000 μg/ml showed maximum scavenging of superoxide radical (87.17) by riboflavin-NBT-system, followed by scavenging of stable radical 1,1-diphenyl-2-picryl hydrazyl radical (83.48%), nitric oxide radical (74.09%) hydroxyl radical (60.96%) at the same concentration. However the extract showed only moderate activity by iron chelation (68.26%). That could be due to higher phenolic content in the extract. This finding suggests that hydro alcoholic extract of *A. indica* possess potent *in vitro* antioxidant activity as compared to the standard ascorbic acid. The results justify the therapeutic applications of the plant in the indigenous system of medicine, augmenting its therapeutic value.

Free radical induced by peroxidation causes development of degenerative diseases[1]. The most critical factor for many diseases is oxidative stress[2]. Free radicals are involved in several pathological conditions such as atherosclerosis, liver disorders, diabetes and nephrotoxicity[3]. Free radicals inactivate enzymes and damage important cellular components causing tissue injury through covalent binding and lipid peroxidation[4]. In our body natural antioxidants such as catalase, superoxide dismutase, and glutathione peroxidase are present while synthetic antioxidant like butylated hydroxyl toluene and butylated hydroxyl anisole are suspected to be carcinogenic and hence no more in use. Therefore, in recent years the search of antioxidant from natural origin has been greatly felt[5]. Free radical scavenger may resist the oxidative stress by quenching the free radicals, inhibiting the free radicals, inhibiting the lipid peroxidation and can prevent diseases[6]. Flavonoids are also reported to possess antioxidant potency[7,8].

*Alocasia indica* (*Araceae*) has been used traditionally in jaundice, diseases of abdomen, spleen, inflammation[9]. The juice of the leaves of the plant is used as digestive, laxative, diuretic, astringent and traditionally used for the treatment of rheumatic arthritis[10]. It has antifungal properties[11]. This plant was found to contain flavonoids, cynogenetic glycosides, alocasin, amino acids, succinic acid and β-lectines[12]. However no reports were published that show the antioxidant potential of the plant. Hence, it was our intention to investigate the antioxidant activity of the leaves of *Alocasia indica.*

All chemicals and solvents were of analytical grade and obtained from HiMedia Chemicals, Mumbai. 1,1-diphenyl-2-picryl hydrazyl (DPPH), was obtained from Sigma Chemicals, USA. The other chemicals used were sodium nitroprusside, sulphanilamide, o-phenanthroline, o-phosphoric acid, napthyl ethylene diamine dihydrochloride, trichloroacetic acid (TCA), nitroblue tetrazolium (NBT), ethylene diamine tetra acetic acid (EDTA), ammonium molybdate, riboflavin, Fe-EDTA, haemoglobin solution which was obtained from Pathology Department, Ayurvedic Medical College Sangali, ascorbic acid used was of analytical grade and procured from Loba Chemicals Ltd., Mumbai. UV/Vis spectrophotometer (Shimadzu 1700) was used for recording the spectra.

Fresh leaves of *A. indica* were collected from different places of Karad, Satara district, India. The plant and leaves were authenticated at the Botany Department, Yashwantrao Chavan College of Science, Karad, India. The so collected fresh leaves were dried in sun light and powdered. Two hundred and fifty grams of the powder was extracted with a mixture of ethanol (95%) and water (1:1) at room temperature by the cold maceration method[13]. The filtrate was collected and concentrated on heating mantle at 45° till a syrupy mass was obtained. Then the extract was further dried in a rotary evaporator and in sunlight. The percent yield was found to be 2.56 with respect to the initial dried plant material. The dried extract was powdered and used for preparation of final concentrations to evaluate antioxidant potential. A preliminary phytochemical screening of the hydroalcoholic extract of *Alocasia indica* was carried out[14]. The standard and test extracts in the dose range of 200-1000 μg/ml were evaluated for antioxidant activity by various *in vitro* models[15–17] with ascorbic acid as the standard.

To 1 ml of the extract of different concentrations, 1 ml solution of DPPH (0.1 mM) was added. An equal amount of methanol and DPPH solution served as control. After 20 min of incubation in the dark, absorbance was measured at 517 nm[18,19]. The experiment was performed in triplicate and the percentage scavenging was calculated.

Nitric oxide was generated from sodium nitroprusside and measured by Griss reaction[20,21]. Sodium nitoprusside (5 mM) in standard phosphate buffer saline solution (0.025 M, pH 7.4) was incubated with different concentrations of (200-1000 μg/ml) of the hydro alcoholic extract dissolved in phosphate buffer saline (0.025 M, pH 7.4) and the tubes were incubated at 25° for 5 h. Control experiments without test compounds but with equivalent amount of buffer were conducted in identical manner. After 5 h, 0.5 ml of solution was removed and diluted with 0.5 ml of Griss reagent (2 g of 1% sulphanilamide, 5 ml of 2% o-phosphoric acid, and 2 g of 0.1% napthyl ethylene diamine dihydrochloride). The absorbance of chromophore formed during diazotization of nitrite with sulphanilamide and its subsequent coupling with napthyl ethylene diamine was read at 546 nm. The experiment was performed in triplicate.

Extracts of different concentrations (200-1000 μg/ml) were taken in different test tubes and evaporated on water bath. To these, 1 ml of Fe-EDTA, 0.5 ml of EDTA and 1 ml DMSO were added and the reaction was initiated by adding 0.5 ml ascorbic acid to each of the test tubes. Test tubes were capped tightly and heated on water bath at 80-90° for 15 min. Then the reaction was terminated by addition of 1 ml of ice-cold TCA (17.5%, w/v) to all the test tubes and kept aside for 5 min. The formaldehyde formed was determined by adding 3 ml Nash reagent (75 g ammonium acetate, 3 ml glacial acetic acid, 2 ml acetyl acetone was mixed and made up to 1000 ml with distilled water). This reaction mixture was kept aside for 15 min for color development[22]. Intensity of the yellow color formed was measured spectrophotometrically at 412 nm against a reagent blank. Percentage scavenging was calculated by comparison of the result of the samples, standard, with that of the blank[23].

The assay was based on the capacity of the sample to inhibit blue formazon formation by scavenging the superoxide radicals generated in the riboflavin-NBT-system. The reaction mixture contains 50 mM phosphate buffer pH 7.6, 20 g riboflavin, 12 mM NBT. Reaction was started by illuminating the test samples of the extract (200-1000 μg/ml). The absorbance was measured at 590 nm. Ascorbic acid was used as positive control[24,25].

One millilitre of each extract (200-1000 μg/ml) was treated with an equivalent amount of reaction mixture which contains 1 ml 0.05% o-phenanthroline in methanol, 2 ml ferric chloride (200 mM). The treated compound was incubated at ambient temperature for 10 min and the absorbance of same was measured at 510 nm. The experiment was performed in triplicate[26,27]. One millilitre of extract of different concentrations (200-1000 μg/ml) was treated with 1 ml of reagent solution (0.6 M sulphuric acid, 28 mM sodium phosphate and 4 mM ammonium molybdate) in an eppendorf tube. The tubes were capped and incubated in thermal block at 95° for 90 min. After cooling to room temperature the absorbance was measured at 695 nm against blank. Ascorbic acid was used as a standard[28].

The degree of glycosylation of haemoglobin *in vitro* can be measured colorimetrically. Haemoglobin (5 g/100 ml) in 0.01 M phosphate buffer pH 7.4 was incubated in presence of 0.02 g/ml concentration of glucose for 72 h in order to find out the best condition for haemoglobin glycosylation. One millilitre of extract of different concentrations (200-1000 μg/ml), 1 ml of glucose solution, 1 ml of gentamycin (20 mg/ml) in 0.01 M phosphate buffer pH 7.4 was incubated in dark at room temperature. Then the glycosylation conc. of compound and their absorbance was measured colorimetrically[29,30].

To make a semi-quantitative visualization possible, hydroalcoholic extract of *A. indica* was applied on a TLC plate and developed in solvent system consisting of ethyl acetate:formic acid:glacial acetic acid:water (14.28:1.42:1.42:2.85 v/v/v/v). The plate was then dipped in a 0.2% solution of DPPH in methanol[31]. The percentage scavenging of each extract in different concentrations for different *in vitro* models are calculated using the following formula, % Scavenging = (Absorbance of standard-absorbance of extract)/(absorbance of standard) × 100, IC_50_ was calculated by using formulae, b= Σxy/Σx^2^, a= 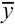
−b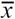 and IC_50_ = a+b(50), where, b is the regression coefficient of x on y; a is the intercept of the line; x is the concentration; and y is % scavenging; 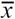 = mean of the concentration; 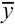 = mean of % scavenging.

The preliminary phytochemical investigation of the hydroalcoholic extract of *Alocasia indica* showed that it contains flavonoids, cynogenetic glycosides, citric acid, ascorbic acid, polyphenolic compounds. Five concentrations ranging from (200-1000 μg/ml) of the hydroalcoholic extract of *A. indica* were tested for their antioxidant potential in different *in vitro* models. It was observed that free radicals were scavenged by test compounds at different concentrations. The antioxidant model and % scavenging of each concentration of extract and standard are shown in Table 1.

**TABLE 1:** ANTIOXIDANT POTENTIAL OF HYDROALCOHOLIC EXTRACT OF *A. INDICA*

Different antioxidant models	% Scavenging at different concentration (μg/ml) [values are mean of 3 replicates]							
	
	Standard AA*(μg/ml)	Test extract: *A. indicia.*						
		
	200	50	100	200	400	800	1000	IC_50_
DPPH**	78.6	60.12	72.52	78.52	81.29	83.46	83.48	9.15
Nitric oxide	64.73	36.98	53.54	63.48	71.59	73.51	74.09	9.69
Hydroxyl radical	52.34	46.27	52.17	56.15	57.23	58.28	60.96	10.17
Superoxide radical	85.06	76.15	82.19	85.7	85.84	86.84	87.17	9.16
Iron chelating activity	63.63	46.31	53.29	59.13	64.52	68.24	68.26	8.68
Total antioxidant capacity	69.56	45.85	53.67	60.86	65.85	69.55	69.56	8.75
Glycosylation of hemoglobin	70.45	62.25	65.15	67.89	68.9	69.67	71.34	10.78

* Ascorbic acid

** 1,1-diphenyl-2-picryl hydrazyl

The maximum % scavenging of each concentration of extract is determined by Riboflavin-NBT- system and DPPH assay as compared to other *in vitro* models. The maximum inhibitory concentration (IC_50_) in all models viz., DPPH, nitric oxide radical, superoxide, iron chelating, total antioxidant capacity, non-enzymatic glycosylation of haemoglobin, hydroxyl radical scavenging activity were found to be 9.15, 9.69, 9.16, 8.675, 8.675, 10.78, 10.17 μg/ml, respectively.

The extract showed better activity in quenching nitric oxide radical with an IC_50_ value 9.69 μg/ml and DPPH radicals with an IC_50_ value of 9.15 μg/ml. However the extract also showed encouraging responses in quenching superoxide with IC_50_ value of 9.16 μg/ml. The activity was moderate in remaining antioxidant models. The result shows that *A. indica* has a potent scavenging activity with increasing % inhibition. Chemical entities that can exist separately with one or more unpaired electrons are called as free radicals. Generation of such free radicals can bring about thousands of reactions and thus cause extensive tissue damage. Lipids, proteins and DNA are all susceptible to attack by free radicals[32,33]. Free radical scavengers may offer resistance against oxidative stress by quenching the free radicals.

From the present results it may be postulated that *A. indica* reduces the radical to the corresponding hydrazine when it reacts with hydrogen donor in the antioxidant principle[34]. Superoxide anion is the first reduction product of oxygen[35] which is measured in the terms of inhibition of generation of oxygen. Quenching of NO_2_ radical by the extract may be due to the antioxidant principles in the extract which compete with the oxygen to react with nitric oxide[36] and thus inhibits the generation of nitrite. The hydroxyl radicals formed by oxidation reacts with DMSO to form formaldehyde by Fe^3+^ ascorbic acid system which is used to detect hydroxyl radicals[21]. The appearance of yellow color spots on violet background of TLC was the indirect measure of rapid screening for antioxidant compounds in the extract.

Ortho-substituted phenolic compounds may exert pro-oxidant effect by interacting with iron. o-phenanthroline quantitatively forms complexes with Fe^2+^ which gets disrupted in presence of chelating agents[19]. The hydroalcoholic extract interfered with the formation of ferrous-o-phenanthroline complex, thereby suggesting that the extract has metal chelating activity. The total antioxidant capacity the extract was calculated based on the formation of phosphomolybdenum complex which was measured spectrophotometrically at 695 nm.

Flavonoids have been shown to possess various biological properties related to antioxidant mechanism[13]. Thus, in the present study, the antioxidant potential of *A. indica* may be attributed to the presence of flavonoinds and the other constituents present there in.
